# Supplementary material for: Erucic Acid-Rich Yellow Mustard Oil Improves Insulin Resistance in KK-Ay Mice
Source: Molecules. 2021 Jan 21;26(3):546. doi: 10.3390/molecules26030546 (PMC7864507; doi:10.3390/molecules26030546)
Supplement: Supplementary file 1 [file molecules-26-00546-s001.pdf]

Table S1. Composition of fatty acids in hydrolyzed YMO

fatty acid (%)	YMO
C16:0	2.55
C18:0	1.04
C18:1	24.36
C18:2	8.95
C18:3 n3	10.61
C20:1	10.87
C22:1	36.89
Others	4.73

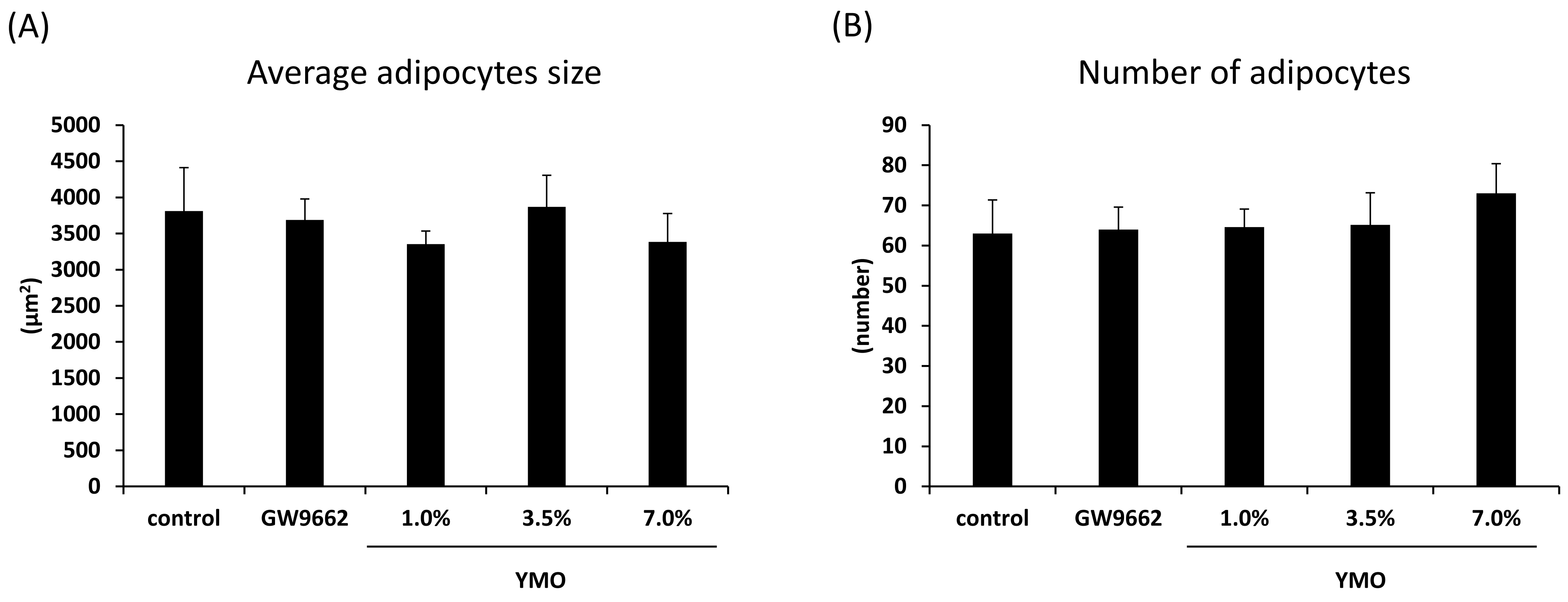

Figure S1. Effect of YMO on adipocytes size and number in perirenal adipose tissue in KK-A^y mice. Perirenal adipose tissue was collected from KK-A^y mice fed with 1.0, 3.5, or 7.0% YMO for 16 weeks. Paraffin sections of adipose tissue were stained with hematoxylin & eosin (HE). (A) Average adipocytes size, (B) Number of adipocytes. Values are mean ± SE, n=7.

Takahashi A et al. Figure S2.

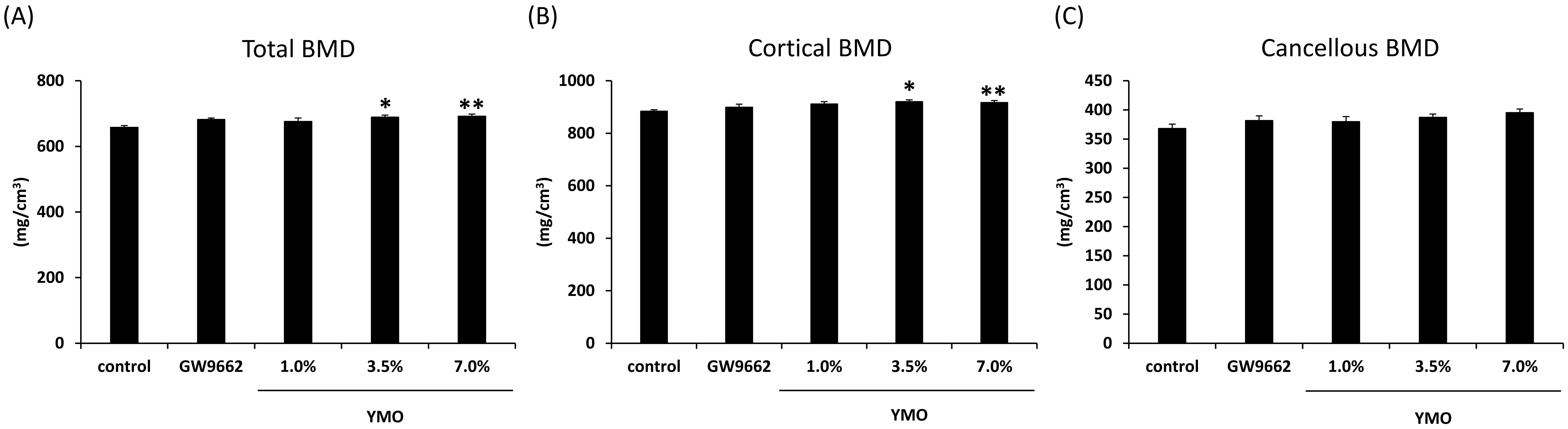

Figure S2. Effect of YMO on bone mineral density (BMD) in KK-A^y mice. Left femur was collected from KK-A^y mice fed with 1.0, 3.5, or 7.0% YMO for 16 weeks. Total BMD (A), cortical BMD (B), and cancellous BMD (C) were measured by peripheral quantitative computed tomography (pQCT) as described in "Materials and Methods." Values are mean ± SE, n=9-11. **$P < 0.01$, *$P < 0.05$ versus control.
